# Predicting arthritis risk with machine learning: Insights from the 2023 National Health Interview Survey data

**DOI:** 10.1371/journal.pone.0336018

**Published:** 2025-11-26

**Authors:** Tianhua Chen, Zhiwei Long

**Affiliations:** Department of Orthopaedics, The Affiliated Longhui People’s Hospital, Shaoyang, China; University of Diyala College of Medicine, IRAQ

## Abstract

Arthritis, a common chronic disease encompassing multiple subtypes of osteoarthritis and rheumatoid arthritis, was explored in this study as a risk-related factor based on data from the 2023 U.S. National Health Interview Survey (NHIS). The study included 26,031 participants (6,849 in the arthritis group; 19,182 in the control group), and 21 variables were found to be significantly different between groups by chi-square test. Fourteen key predictors were screened using support vector machine recursive feature elimination (SVM-RFE): age, general health, chronic obstructive pulmonary disease, gender, hypertension, coronary heart disease, body mass index (BMI), cancer, depression, dementia, asthma, diabetes, smoking status, and hepatitis. The column-linear graphical model constructed based on these variables showed excellent predictive performance (AUC = 0.813), the slope of the calibration curve was close to 1 (P = 0.444) indicating high predictive accuracy, and the decision curve analysis showed that its net benefit was better than that of a single predictor. The study demonstrated that the NHIS column-line graph model constructed based on machine learning algorithms can effectively predict the risk of arthritis and provide an important reference for clinical management. The prediction model established in this study provides a theoretical basis for accurate prevention and treatment strategies for arthritis.

## 1. Introduction

Arthritis is a common chronic disease that includes osteoarthritis (OA), rheumatoid arthritis (RA), psoriatic arthritis, and other subtypes of arthritis, and currently affects more than 500 million people worldwide [1]. Arthritis is characterized by pain, stiffness and functional limitations, which greatly reduces patients’ quality of life and creates a significant socio-economic burden due to disability and high medical costs [2]. OA is often caused by age-related cartilage degeneration and biomechanical stress, whereas RA is caused by autoimmune-mediated synovial inflammation [3,4]. Despite differences in subtypes, they share numerous risk factors and collectively impose a substantial disease burden. Risk factors such as aging, obesity, genetic predisposition, and comorbidities (e.g., hypertension, diabetes mellitus) are associated with its pathogenesis [5]. Current diagnostic methods rely on clinical assessment, imaging, and biomarkers, but early detection remains challenging, and therapeutic strategies often focus on symptom management rather than disease modification [6]. These limitations highlight the need to identify novel risk predictors and develop robust models for early intervention. Given that the NHIS database used in this study queries arthritis as a single category via the question “Have you ever had arthritis?”, the present study aims to explore the common risk factors for arthritis in a broad sense, rather than distinguishing between specific subtypes.

The NHIS is a nationally representative cross-sectional survey that systematically collects comprehensive health data, including demographics, chronic diseases, lifestyle behaviors, and socioeconomic determinants. Its strengths of large sample size (e.g., more than 25,000 participants per year), standardization of protocols, and integration of socioeconomic determinants make it well-suited for studying multifactorial diseases such as arthritis [7–9]. Previous NHIS-based studies have elucidated the relationship between arthritis and obesity, smoking, and cardio-metabolic complications (e.g., hypertension, diabetes), highlighting the role of arthritis in risk stratification [10,11]. In addition, NHIS data inform public health policy by quantifying differences in arthritis prevalence among population subgroups, such as the fact that the prevalence of OA is twice as high in women as in men. However, existing arthritis studies using the NHIS have focused primarily on prevalence estimates or isolated risk associations and lack comprehensive models that assess multifactorial interactions, and these analyses rarely employ advanced feature selection techniques to optimize predictive accuracy [12–14]. This gap highlights the untapped potential of NHIS in developing predictive tools for arthritis risk stratification.

Despite progress in understanding the pathogenesis of arthritis, critical challenges remain. First, most risk prediction models rely on a limited number of variables, neglecting the synergistic effects of comorbidities and lifestyle factors [15]. Second, conventional statistical methods often fail to handle high-dimensional data efficiently, potentially missing important predictors. Recent studies have highlighted the utility of machine learning (ML) algorithms, such as support vector machine-recursive feature elimination (SVM-RFE), in optimizing variable selection from complex datasets [16]. However, few studies have integrated ML techniques with nomogram construction to improve clinical interpretability. Building on this foundation, our study aims to (1) identify critical variables associated with arthritis risk using SVM-RFE and (2) develop a clinically actionable nomogram to quantify individualized risk. By synthesizing demographic, clinical, and behavioral data from NHIS, this work addresses the unmet need for multifactorial risk assessment tools and provides insights for targeted interventions and resource allocation in arthritis management.

## 2. Materials and methods

### 2.1. Data collection

**Fig 1:**
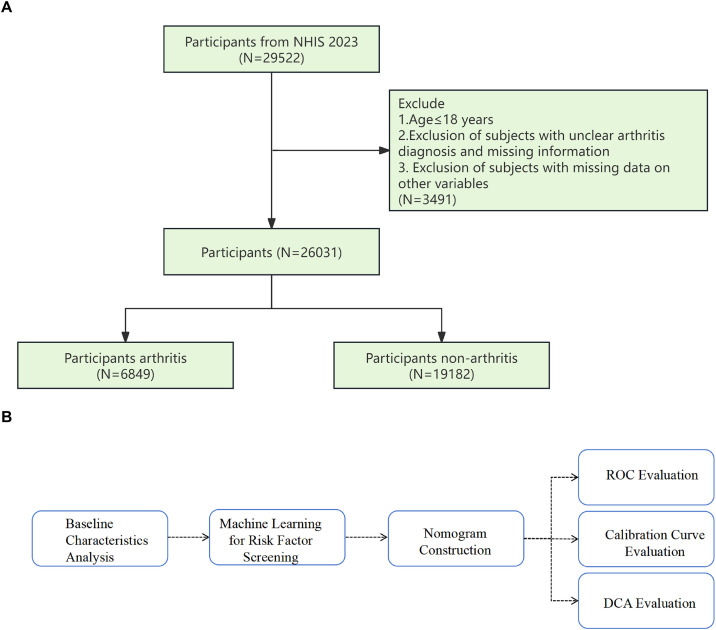
Flow diagram of participant inclusion/exclusion criteria and the analytical workflow of this study. A. Flowchart of Participant Inclusion and Exclusion, B. The overall analysis flow chart.

Data were obtained from the NHIS database (https://www.cdc.gov/nchs/nhis/) (accessed on December 17, 2024), a continuing survey that began in 1957 and documents information on the amount, distribution, and effects of illness and disability in the U.S. population. This study included adult participants with a history of arthritis from the 2023 NHIS data. Samples of participants with arthritis, as identified in the NHIS questionnaire, were selected for this study. The data access date for this study was 2024.12.17. Information identifying individual participants could not be accessed during or after data collection. The exclusion criteria were: (1) excluding participants aged 18 years or younger; (2) excluding participants with unclear arthritis diagnoses or missing information; (3) excluding participants with missing data for other variables. A total of 26,031 participants were recruited (arthritis = 6,849; non-arthritis = 19,182), and the flow chart for exclusion and inclusion was shown in Fig 1A. In addition, a flowchart illustrating the overall analytical framework of this study was also presented (Fig 1B).

### 2.2. Outcome definition

The NHIS database was accessed, the 2023 data was selected, and the Sample Adult Interview was chosen. The codebook system was entered, and ARTHEV_A was searched, where participants were asked, “Have you ever had arthritis?” Those who answered “yes” were categorized as the arthritis group and coded as 1, while those who answered “no” were categorized as the non-arthritis group and coded as 0.

### 2.3. Variables definition

The variables included sociodemographics, health status, and related diseases (S1 Table), as detailed below:

The sociodemographic characteristics included in the study were as follows: age (18–44 years coded as 1, 45–64 years as 2, and 65 years or older as 3); sex (male coded as 1, female as 2); race (Hispanic (Mexican/Mexican American) coded as 1, Hispanic (all other groups) as 2, non-Hispanic as 3); education level (less than high school coded as 1, high school graduate as 2, greater than high school as 3); marital status (married or living with a partner coded as 1, divorced/separated/widowed/never married as 2); poverty status (1–3 coded as 1, 4–7 as 2, and greater than 8 as 3); region of residence (Northeast coded as 1, Midwest as 2, South as 3, and West as 4); smoking status (never smokers coded as 1, former or current smokers as 2).

The health status data were as follows: Body Mass Index (BMI) was classified into 4 categories: underweight (<18.5 kg/m², coded as 1), normal weight (18.5–24.9 kg/m², coded as 2), overweight (25.0–29.9 kg/m², coded as 3), and obesity (30.0–40.0 kg/m², coded as 4); overall health status was classified as healthy (coded as 1) or not very healthy (coded as 2); mental health was defined based on whether the individual had received counseling or treatment from a mental health professional in the past 12 months (received care, coded as 1; did not receive care, coded as 2); health insurance status was categorized as not covered (coded as 1) or covered (coded as 2).

The relevant disease data were include: Diabetes (“ever been diagnosed with diabetes?”, “yes” coded as 1, “no” coded as 2); hypertension (“ever been told you have high blood pressure?”, “yes” coded as 1, “no” coded as 2); cancer (“ever been diagnosed with cancer?”, “yes” coded as 1, “no” coded as 2); asthma (“ever been diagnosed with asthma?”, “yes” coded as 1, “no” coded as 2); chronic obstructive pulmonary disease (“ever been told you have chronic obstructive pulmonary disease, emphysema, or chronic bronchitis?”, “yes” coded as 1, “no” coded as 2); hepatitis (“ever been diagnosed with hepatitis?”, “yes” coded as 1, “no” coded as 2); stroke (“ever been told you had a stroke?”, “yes”coded as 1, “no” coded as 2); dementia (“ever been diagnosed with dementia?”, “yes” coded as 1, “no” coded as 2); coronary artery disease (“ever been told you have coronary artery disease?”, “yes” coded as 1, “no” coded as 2); depression (“ever been told you have depression?”, “yes” coded as 1, “no” coded as 2).

### 2.4. Statistical analysis

A baseline table was constructed based on 22 variables, and the tableone package (v 0.13.2) was used for its generation [17]. Categorical variables were shown as counts (percentages), and the Chi-square test was applied to assess statistical differences between the arthritis group (1) and the non-arthritis group (0) (*P* < 0.05) to identify significant variables. A total of 26,031 recruited participants were divided into a training set and a test set at a ratio of 7:3, with 18,223 samples in the training set and 7,808 samples in the test set for subsequent analysis. Subsequently, based on the variables with significant differences, SVM-RFE was performed via the “caret” package (v 6.0.93) in the training set (10-fold cross-validation), and the variables output at the highest prediction accuracy of the model were selected as key variables [18]. And the model’s F1-score, Recall, Sensitivity, Specificity, Precision, and Area Under the Curve (AUC) (95% CI) were calculated in the test set to further evaluate its predictive performance. Finally, based on the key variables, a nomogram was constructed using the “RMS” package (v 6.5−0) [19] in the training set. Receiver operating characteristic (ROC) analysis was conducted using the “pROC” package (v 1.18.0), decision curve analysis (DCA) was conducted using the “ggDCA” package (v 1.6), and a calibration curve for the nomogram was plotted using the “regplot” package (v 1.1) [17,20,21]. All statistical analyses were performed using R (v 4.2.2).

## 3. Results

### 3.1. Identification of key variables

**Fig 2:**
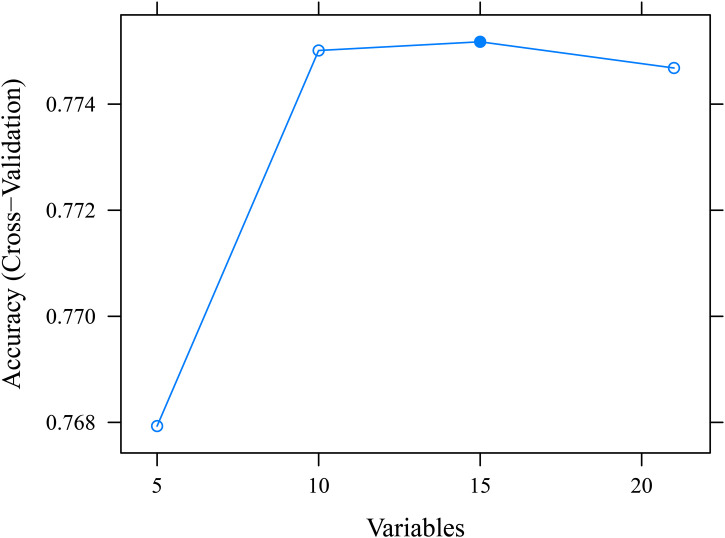
Accuracy of SVM-RFE Algorithm for Feature Selection.

The baseline table results indicated significant differences (*P* < 0.001) between the group 1 and group 2 across 21 variables, including age, poverty status, region of residence, smoking status, BMI, overall health status, sex, race, health insurance status, diabetes, hypertension, cancer, asthma, education level, marital status, chronic obstructive pulmonary disease, hepatitis, stroke, dementia, coronary heart disease, and depression. In the arthritis group, the proportions of participants who were aged 65 and older, female, non-Hispanic, had a high school education or higher, divorced, separated, widowed, or never married, had a poverty status greater than 8, from the Southern region, former or current smokers, obese, healthy, with health insurance coverage, non-diabetic, hypertensive, non-cancerous, non-asthmatic, non-chronic obstructive pulmonary disease, non-hepatitis, without a history of stroke, non-dementia, non-coronary heart disease, and non-depression were relatively higher, with the following respective percentages: 59.4%, 61.3%, 91.6%, 61.2%, 52.3%, 65.6%, 38.0%, 52.2%, 41.0%, 68.5%, 87.1%, 97.7%, 71.4%, 60.3%, 77.5%, 80.6%, 86.8%, 96.2%, 92.4%, 97.3%, 86.8%, and 84.2% (Table 1). A total of 15 key variables were identified by the SVM-RFE machine learning algorithm, including age, overall health status, chronic obstructive pulmonary disease, sex, hypertension, coronary heart disease, BMI, cancer, depression, dementia, asthma, diabetes, poverty status, stroke, and hepatitis (Fig 2). The evaluation results of the model in the test set were AUC 0.751 (95% CI 0.738–0.764), F1 0.425, Recall/Sensitivity 0.320, Specificity 0.934, and Precision 0.635, indicating that the model had good predictive performance (Table 2).

**Table 1:** Baseline table.

	level	0	1	p
n		19182	6849	
age (%)	1	9043 (47.1)	604 (8.8)	<0.001
	2	5831 (30.4)	2179 (31.8)	
	3	4308 (22.5)	4066 (59.4)	
sex (%)	1	9333 (48.7)	2648 (38.7)	<0.001
	2	9849 (51.3)	4201 (61.3)	
race (%)	1	1793 (9.3)	295 (4.3)	<0.001
	2	1409 (7.3)	282 (4.1)	
	3	15980 (83.3)	6272 (91.6)	
education level (%)	1	1366 (7.1)	757 (11.1)	<0.001
	2	4626 (24.1)	1902 (27.8)	
	3	13190 (68.8)	4190 (61.2)	
marital status (%)	1	10523 (54.9)	3267 (47.7)	<0.001
	2	8659 (45.1)	3582 (52.3)	
poverty status (%)	1	1754 (9.1)	834 (12.2)	<0.001
	2	3196 (16.7)	1521 (22.2)	
	3	14232 (74.2)	4494 (65.6)	
region of residence (%)	1	2957 (15.4)	1055 (15.4)	<0.001
	2	4093 (21.3)	1656 (24.2)	
	3	7071 (36.9)	2602 (38.0)	
	4	5061 (26.4)	1536 (22.4)	
smoking status (%)	1	6253 (32.6)	3277 (47.8)	<0.001
	2	12929 (67.4)	3572 (52.2)	
BMI (%)	1	301 (1.6)	97 (1.4)	<0.001
	2	6293 (32.8)	1640 (23.9)	
	3	6701 (34.9)	2305 (33.7)	
	4	5887 (30.7)	2807 (41.0)	
overall health status (%)	1	17183 (89.6)	4690 (68.5)	<0.001
	2	1999 (10.4)	2159 (31.5)	
mental health (%)	1	2594 (13.5)	882 (12.9)	0.184
	2	16588 (86.5)	5967 (87.1)	
health insurance status (%)	1	1595 (8.3)	155 (2.3)	<0.001
	2	17587 (91.7)	6694 (97.7)	
Diabetes (%)	1	2669 (13.9)	1961 (28.6)	<0.001
	2	16513 (86.1)	4888 (71.4)	
hypertension (%)	1	5707 (29.8)	4128 (60.3)	<0.001
	2	13475 (70.2)	2721 (39.7)	
cancer (%)	1	1853 (9.7)	1544 (22.5)	<0.001
	2	17329 (90.3)	5305 (77.5)	
asthma (%)	1	2526 (13.2)	1332 (19.4)	<0.001
	2	16656 (86.8)	5517 (80.6)	
chronic obstructive pulmonary disease (%)	1	510 (2.7)	907 (13.2)	<0.001
	2	18672 (97.3)	5942 (86.8)	
hepatitis (%)	1	281 (1.5)	259 (3.8)	<0.001
	2	18901 (98.5)	6590 (96.2)	
stroke (%)	1	461 (2.4)	522 (7.6)	<0.001
	2	18721 (97.6)	6327 (92.4)	
dementia (%)	1	141 (0.7)	185 (2.7)	<0.001
	2	19041 (99.3)	6664 (97.3)	
coronary artery disease (%)	1	745 (3.9)	905 (13.2)	<0.001
	2	18437 (96.1)	5944 (86.8)	
depression (%)	1	1690 (8.8)	1082 (15.8)	<0.001
	2	17492 (91.2)	5767 (84.2)	

**Table 2:** Evaluation model indicators of test set.

AUC	AUC_CI_low	AUC_CI_mid	AUC_CI_high	F1	Recall	Sensitivity	Specificity	Precision
0.751221156342195	0.738302789807174	0.751221156342195	0.764139522877215	0.425380382000647	0.319863680623174	0.319863680623174	0.934306569343066	0.634782608695652

### 3.2. Establishment of a robust nomogram model based on key variables

**Fig 3:**
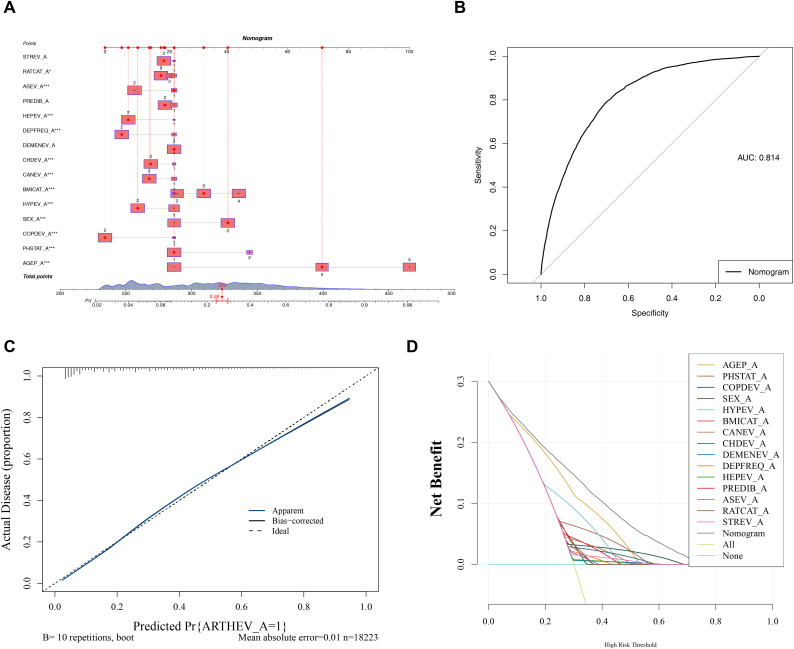
Construction and validation of the nomogram. A. Nomogram, B. ROC Curve, C. Calibration Curve, D. DCA Decision Curve.

To further predict the occurrence of arthritis, a nomogram was constructed based on the key variables (Fig 3A). The ROC curve revealed an AUC value of 0.814 for the nomogram (Fig 3B). The calibration curve was then evaluated and the slope approaching 1 indicated that the model’s predictions were highly accurate (Fig 3C). Finally, the DCA results of this study showed that within the full risk threshold range (0–1) indicated by the abscissa, both the nomogram curve and the prediction curves corresponding to each single risk factor were located in the area above the “All” line and “None” line, with no cases of falling below the reference lines. Additionally, it was found that the net benefit value of the nomogram model curve was significantly higher than that of most single risk factor curves. This indicated that the gain of a single risk factor for clinical decision-making was limited, while the nomogram model integrated with multiple factors had better decision-making guidance value (Fig 3D). These results suggested that the nomogram based on the key variables demonstrated excellent predictive performance for arthritis.

## 4. Discussion

Arthritis, a leading cause of disability globally, is influenced by a complex interplay of demographic, socioeconomic, and comorbid factors [22]. Leveraging data from the NHIS, this study systematically evaluated 22 variables spanning sociodemographic characteristics, health behaviors, and chronic conditions to identify key predictors of arthritis. Through baseline comparisons and machine learning-based variable selection, 15 critical variables were identified, including age, sex, hypertension, and chronic obstructive pulmonary disease (COPD). A nomogram integrating these variables showed robust predictive performance (AUC = 0.814), outperforming individual predictors. These findings highlight the multifactorial nature of arthritis risk and provide a data-driven tool for individualized risk stratification, consistent with current efforts to optimize early diagnosis and resource allocation in arthritis management [23,24].

The present study found that the arthritis cohort had a higher proportion of older age (≥65 years) and females (59.4% and 61.3%, respectively), which is consistent with previous findings. Aging is strongly associated with cartilage degeneration and systemic inflammation, while hormonal differences, particularly the role of oestrogen in immune modulation, may explain the sex differences [24–26]. Notably, the higher prevalence of hypertension (71.4%) and coronary heart disease (CHD, 86.8%) in arthritis patients aligns with evidence linking chronic inflammation to cardiovascular comorbidities. Pro-inflammatory cytokines such as TNF-α and IL-6, which are elevated in arthritis, accelerate endothelial dysfunction and atherosclerosis, creating a bidirectional relationship between arthritis and cardiovascular pathologies [25,27]. Similarly, obesity (BMI ≥ 30, 41.0%) contributes to mechanical joint stress and adipose-derived inflammation via adipokines like leptin, further supporting its role in arthritis pathogenesis [28].

COPD and hepatitis-factors less conventionally emphasized in arthritis research. The inclusion of COPD (77.5%) and hepatitis (96.2% non-hepatitis) as predictors underlines the systemic inflammatory cross-talk. Common mechanisms, such as neutrophil extracellular traps (NETs) in COPD and autoimmune dysregulation in viral hepatitis, may amplify joint inflammation [29]. Conversely, the lower prevalence of diabetes (60.3% non-diabetic) contrasts with some studies linking hyperglycemia to osteoarthritis progression. This discrepancy may reflect differences in population characteristics or confounding by antidiabetic therapies with anti-inflammatory properties.

The strong predictive ability of the nomogram (AUC: 0.814) outperforms single variable models, highlighting the value of multivariate risk assessment. This is consistent with prior studies advocating integrated models for chronic disease prediction [30]. For instance, a similar nomogram for rheumatoid arthritis achieved an AUC of 0.79 by incorporating age, BMI, and smoking [31]. Our model extends this by integrating understudied variables like hepatitis and dementia, thereby increasing granularity. Clinically, such tools allow for personalised risk quantification, supporting early intervention, e.g., targeting smoking cessation in high-risk obese individuals or intensifying comorbidity management in hypertensive patients. Additionally, the calibration slope close to 1 suggests reliability across risk strata, supporting its utility in diverse populations.

Decision curve analysis (DCA) further validates the clinical relevance of the nomogram, demonstrating superior net benefit across threshold probabilities. This is in contrast to traditional biomarkers like CRP or ESR, which lack specificity for arthritis [32]. However, the model’s reliance on self-reported NHIS data may introduce recall bias, a limitation mitigated by the survey’s rigorous validation protocols.

In conclusion, this study leveraged the NHIS database and machine learning approaches to identify 15 critical variables-including age, sex, poverty status, and comorbidities-that collectively inform arthritis risk stratification. The robust nomogram model (AUC = 0.814) not only highlights the multifactorial nature of arthritis but also provides a novel framework for individualized risk assessment, advancing both mechanistic understanding and clinical management. However, several limitations must be acknowledged. The cross-sectional design limits causal interpretation, and reliance on self-reported diagnoses may underestimate true prevalence. Additionally, the absence of biomarker or genetic data restricts insight into underlying pathways. The lack of stratification by arthritis subtypes in this study may hinder researchers from precisely identifying the key pathogenic factors for each subtype, thereby impeding the formulation of targeted preventive strategies at the level of the underlying etiology. Furthermore, due to data limitations, we were unable to distinguish between patients groups with arthritis and those with joint pain, which may have led to an overestimation of the inflammatory burden in the results. Moreover, although the statistical analysis methods employed in this study can effectively validate the predictive value of risk factors, they exhibit significant limitations in terms of assumptions regarding variable associations, validation scenarios, data processing, and causal inference. Future studies could further enhance the scientific rigor and clinical applicability of the model by incorporating nonlinear regression models, conducting external multi-center validations, and adopting causal inference methodologies. Future studies integrating longitudinal designs and multi-omics data may further refine predictive accuracy and biological relevance.

## 5. Conclusion

This machine learning study identifies 14 arthritis predictors (established/novel) to develop a clinical nomogram, emphasizing multivariable modeling for precision medicine and comorbidity management. Although limited by cross-sectional data, it provides a framework for early intervention. Future research requires longitudinal validation and mechanistic exploration of predictor interactions to refine prevention strategies.

## Supporting information

S1 Table Variable and its number information. (DOCX)
