# Supplementary material for: Predicting arthritis risk with machine learning: Insights from the 2023 National Health Interview Survey data
Source: PLoS One. 2025 Nov 26;20(11):e0336018. doi: 10.1371/journal.pone.0336018 (PMC12654921; doi:10.1371/journal.pone.0336018)
Supplement: S1 Table — (DOCX) [file pone.0336018.s001.docx]

| Variables | Definition Codes | Year |
| --- | --- | --- |
| age | AGEP_A | 2023 |
| sex | SEX_A | 2023 |
| race | HISDETP_A | 2023 |
| education level | EDUCP_A | 2023 |
| marital status | MARITAL_A | 2023 |
| poverty status | RATCAT_A | 2023 |
| region of residence | REGION | 2023 |
| smoking status | SMKCIGST_A | 2023 |
| BMI | BMICAT_A | 2023 |
| overall health status | PHSTAT_A | 2023 |
| mental health | MHTHRPY_A | 2023 |
| health insurance status | NOTCOV_A | 2023 |
| Diabetes | PREDIB_A | 2023 |
| hypertension | HYPEV_A | 2023 |
| cancer | CANEV_A | 2023 |
| asthma | ASEV_A | 2023 |
| chronic obstructive pulmonary disease | COPDEV_A | 2023 |
| hepatitis | HEPEV_A | 2023 |
| stroke | STREV_A | 2023 |
| dementia | DEMENEV_A | 2023 |
| coronary artery disease | CHDEV_A | 2023 |
| depression | DEPFREQ_A | 2023 |
